# An Unusual Case of Rising Serum βhCG in the Setting of Retained Products of Conception: A Case Report

**DOI:** 10.7759/cureus.104967

**Published:** 2026-03-10

**Authors:** Kira Sklar

**Affiliations:** 1 Obstetrics and Gynaecology, The Sutherland Hospital, Sydney, AUS

**Keywords:** gynaecology and obstetrics, pregnancy of unknown location, retained products of conception (rpoc), serum beta hcg, surgical hysteroscopy

## Abstract

Retained products of conception (RPOC) is defined by persistent trophoblastic tissue inside the uterine cavity. This can be present after a vaginal or cesarean birth, miscarriage, or termination. Patients with RPOC often present with abnormal or persistent bleeding per vagina, abnormal vaginal discharge, or pain. The presence of RPOC can be associated with a positive serum beta human chorionic gonadotropin hormone (βhCG), which is expected to remain stagnant until resolution of the RPOC. This case report outlines a presentation wherein an inappropriately rising βhCG was found to be attributed to RPOC. This diagnosis was confirmed by histopathology collected during surgical management.

## Introduction

Retained products of conception (RPOC) is defined by persistent trophoblastic tissue inside the uterine cavity after pregnancy [1]. This can occur after vaginal or cesarean birth, or after a miscarriage or termination. Patients with RPOC often present with persistent bleeding per vagina, pain, or infection. Following a miscarriage, RPOC is estimated to occur in up to 6% of women [1]. Ultrasound can be useful in identifying RPOC if it demonstrates a thickened endometrial echo complex or a discrete mass in the uterine cavity [2]. When RPOC are present, there can be an associated positive beta human chorionic gonadotropin hormone (βhCG), which is expected to remain stagnant until resolution of the RPOC [3]. The differential diagnosis of a rising βhCG with no confirmed intrauterine pregnancy includes an extrauterine pregnancy or gestational trophoblastic disease. This case study explores an unusual presentation wherein an inappropriately rising βhCG was shown to be associated with RPOC following a miscarriage, and the histopathological diagnosis excluded gestational trophoblastic disease.

## Case presentation

A 37-year-old G6P1 woman was seen in the Emergency Department at 5 weeks of gestation after bleeding per vagina in the setting of an IVF pregnancy of unknown location (PUL). She had a background of two previous ectopic pregnancies, which were both managed surgically. She had also had two previous miscarriages managed conservatively.

The patient’s initial βhCG in the Emergency Department was 3,153 mIU/mL. She had an ultrasound, which demonstrated no intrauterine or extrauterine pregnancy. The impression was a nonviable PUL, and she was referred to the Early Pregnancy Assessment Service (EPAS) for follow-up and ongoing βhCG tracking.

The patient repeated her βhCG after 48 hours, and it was shown to decrease to 527 mIU/mL. Given both the significant decrease and patient preference, she proceeded to follow up with βhCG tracking for expectant management. After a further 48 hours, the βhCG decreased to 90 mIU/mL (Table 1).

**Table 1:** Serum βhCG trend from initial presentation up to decision for D&C In this laboratory, a serum βhCG is positive when >5 mIU/mL. βhCG: beta human chorionic gonadotropin hormone; D&C: dilation and curettage

Day (from initial presentation)	Serum βhCG (mIU/mL)
0	3153
2	527
7	90
14	144
15	171
17	191
19	222
21	243

The patient, who remained pain-free, then repeated her βhCG one week later, and it had increased to 144 mIU/mL. She had another βhCG collected 24-48 hours later, which was 171 mIU/mL, and she was advised to repeat a pelvic ultrasound and attend for assessment, with the impression of a suspected ectopic pregnancy.

**Figure 1:**
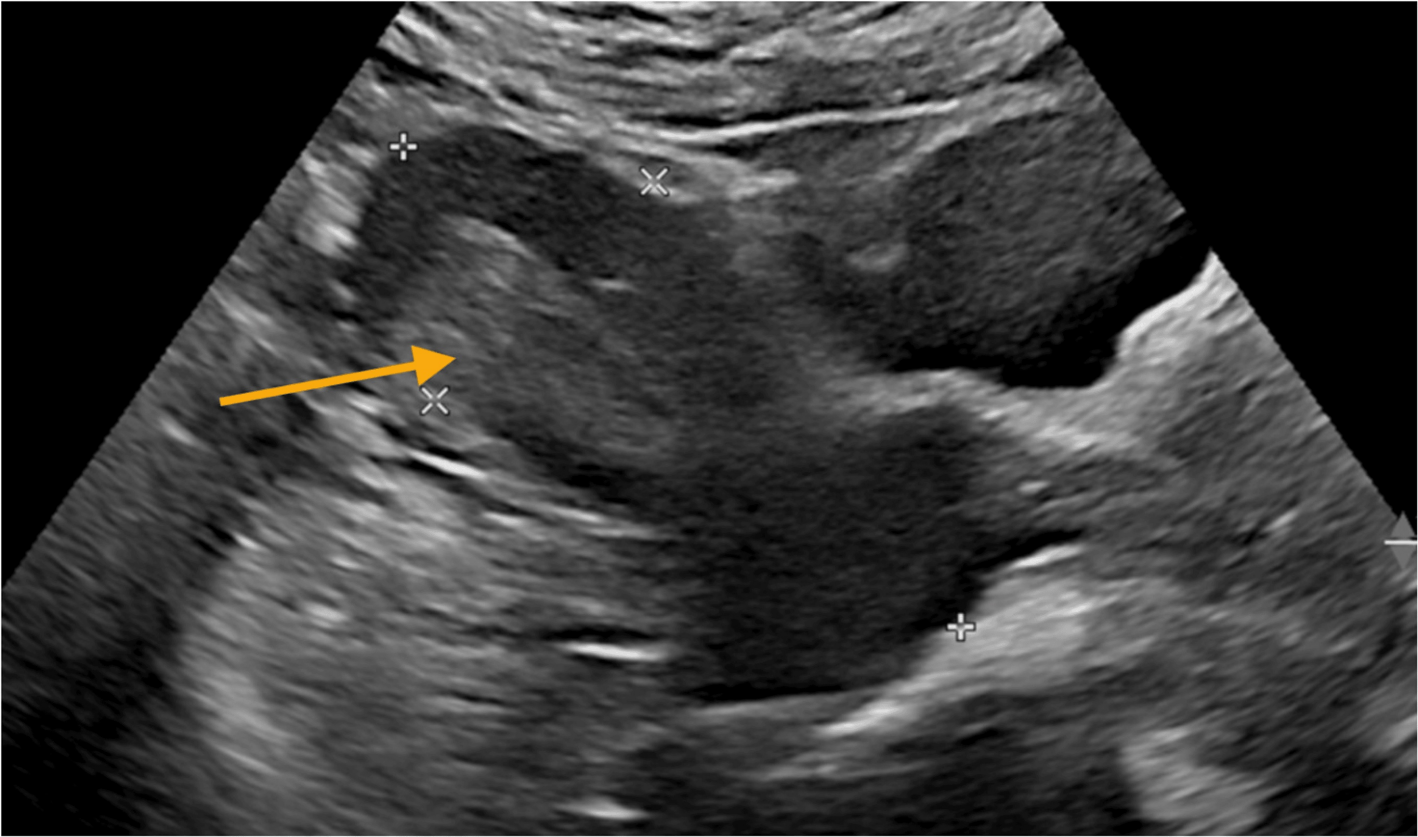
Ultrasound appearance of retained products of conception The associated serum βhCG was 243 mIU/mL.

The patient’s repeat pelvic ultrasound demonstrated heterogeneous material with vascularity in the endometrial cavity, consistent with retained products of conception (Figure 1). The findings were discussed with the patient, including the diagnostic uncertainty, given that a rising βhCG is not typically associated with retained products. Some other possible diagnoses, including an ectopic pregnancy, a heterotopic pregnancy (with an intrauterine miscarriage and RPOC), or a molar pregnancy, were discussed with the patient at this stage.

The patient was offered a hysteroscopic resection of retained products with ultrasound-guided dilation and curettage (HD&C) for tissue sampling, as well as concurrent methotrexate to cover for a possible ectopic pregnancy. The patient’s preference was to avoid methotrexate in the first instance, but she was agreeable to the surgical procedure.

**Figure 2:**
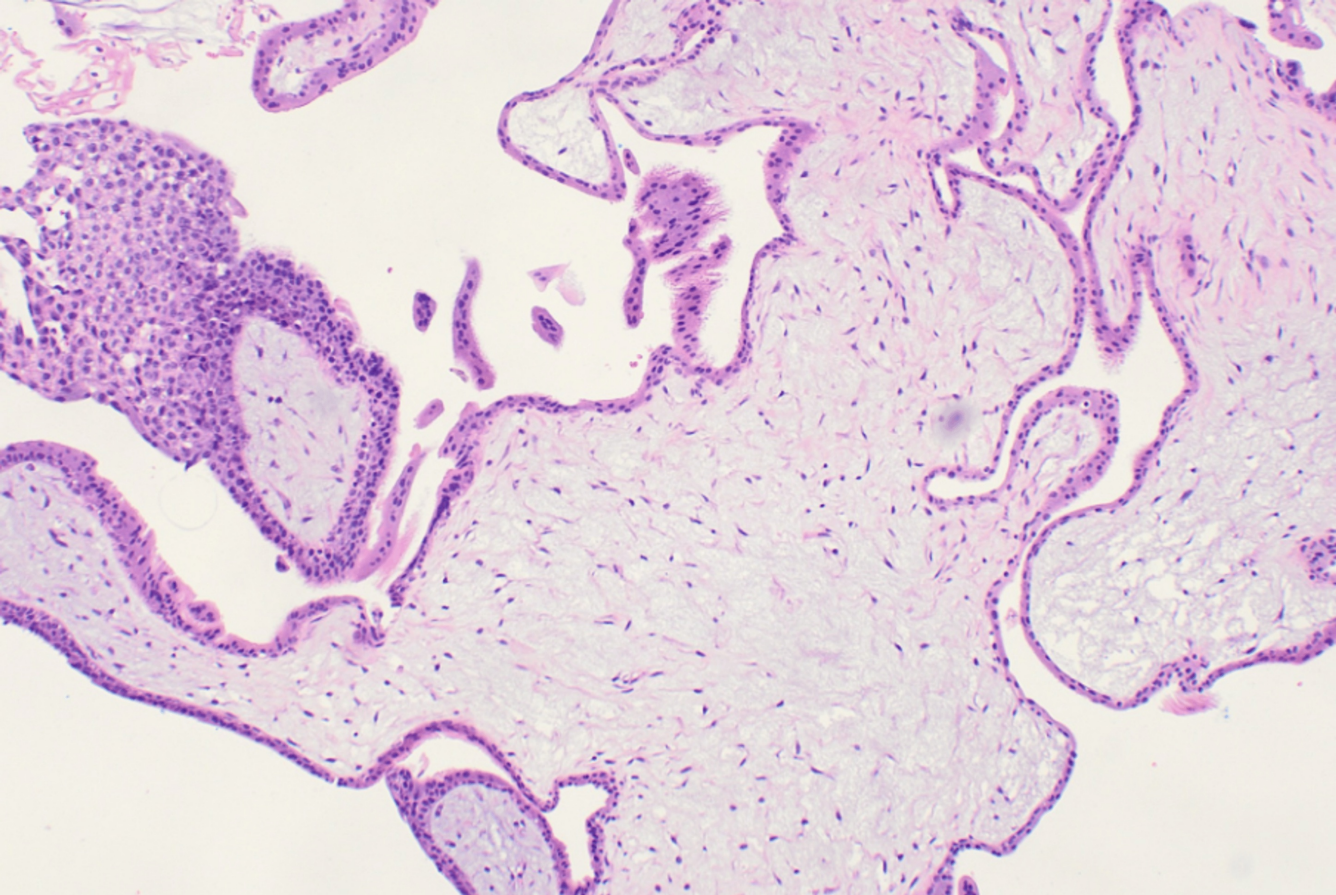
Histopathology of chorionic villi The villi are of the expected size, with no edema, cistern formation, or trophoblastic hyperplasia.

The patient proceeded to have an HD&C, during which suspected calcified retained products of conception were seen. Histopathology confirmed products of conception with no unusual features to suggest molar pregnancy (Figure 2). Her βhCG was repeated one week postoperatively and was 15 mIU/mL. After one further week, her βhCG was less than one (i.e., negative). She was clinically well with no further bleeding and was discharged from EPAS.

## Discussion

Serum βhCG typically follows a predictable trend in early pregnancy as well as following delivery, miscarriage, or termination of pregnancy. In the first trimester of a viable intrauterine pregnancy, serum βhCG approximately doubles every 48 hours [4]. When the serum βhCG does not follow this pattern, a pregnancy is suspected to be nonviable. While ultrasound imaging is the mainstay of early pregnancy diagnosis, an intrauterine gestational sac is usually not visualized on ultrasound prior to the βhCG reaching 1500 to 2000 mIU/mL, hence the utility in tracking the βhCG trend [5,6]. A positive serum βhCG with a nondiagnostic ultrasound constitutes a PUL [5]. Serial serum βhCG is commonly used to follow up on a PUL until a diagnosis is established [6]. Resolution of a nonviable pregnancy is associated with a falling serum βhCG, and once the value is negative, it is considered resolved [7].

Retained products of conception are a common complication of miscarriage [2]. RPOC is suspected if a woman has persistent bleeding or pain following birth, miscarriage, or termination, and an ultrasound can assist with diagnosis [2]. A positive βhCG can support the diagnosis and is expected to be stagnant until resolution of the RPOC [3,7]. βhCG is not always involved in the diagnosis of RPOC, and cannot be used in isolation for diagnosis, however [8]. A serum βhCG is expected to demonstrate a rapid decline following pregnancy loss or termination, and following surgical evacuation of RPOC, the serum βhCG is expected to decrease by at least 15% in the subsequent 24 hours [6,8].

This case demonstrated an unusual presentation wherein the serum βhCG initially had a significant drop, followed by a slow, inappropriate rise, which was not consistent with a viable intrauterine pregnancy. Given the rising βhCG, an ectopic pregnancy was suspected initially; however, a progress ultrasound seemed to suggest RPOC. Hysteroscopic resection findings supported this ultrasound. Gestational trophoblastic disease was also considered; however, histopathology results confirmed the diagnosis of RPOC with no atypical features to suggest gestational trophoblastic disease. The patient then had a serial βhCG following surgery, which decreased by greater than 15%, consistent with intrauterine RPOC [6].

It is unclear why RPOC was associated with an increasing serum βhCG in this case, seeing that RPOC is nonviable tissue. Given that the diagnosis was unclear with differentials including an ectopic pregnancy or gestational trophoblastic disease, gaining tissue histopathology was essential for confirming the unexpected diagnosis.

## Conclusions

A PUL poses a clinical challenge in that diagnostic uncertainty makes it difficult to select appropriate management. There are many differential diagnoses to consider with an inappropriately rising βhCG, and typically, RPOC is not consistent with this type of presentation.

This report illustrates a case where RPOC after an intrauterine miscarriage was found to be the cause of an inappropriately rising βhCG. While ultrasound, histopathology, and postoperative βhCG tracking together confirmed this diagnosis, the mechanism underlying how nonviable tissue contributed to a rising βhCG is not clear. Further research to understand why this could occur would be useful.
